# Feto-Maternal Microchimerism: The Pre-eclampsia Conundrum

**DOI:** 10.3389/fimmu.2019.00659

**Published:** 2019-03-29

**Authors:** Sinuhe Hahn, Paul Hasler, Lenka Vokalova, Shane Vontelin van Breda, Nandor Gabor Than, Irene Mathilde Hoesli, Olav Lapaire, Simona W. Rossi

**Affiliations:** ^1^Department of Biomedicine, University Hospital Basel, Basel, Switzerland; ^2^Division of Rheumatology, Medical University Department, Kantonsspital Aarau, Aarau, Switzerland; ^3^Systems Biology of Reproduction Lendulet Research Group, Institute of Enzymology, Research Centre for Natural Sciences, Hungarian Academy of Sciences, Budapest, Hungary; ^4^Department of Obstetrics, University Women's Hospital Basel, Basel, Switzerland

**Keywords:** feto-maternal microchimerism, pre-eclampsia, non-inherited-maternal-antigens, cell-free DNA, autoimmunity

## Abstract

Feto-maternal microchimerism (FMM) involves bidirectional cross-placental trafficking during pregnancy, leading to a micro-chimeric state that can persist for decades. In this manner a pregnant woman will harbor cells from her mother, as well as, cells from her child. Historically, eclampsia, a severe disorder of pregnancy provided the basis for FMM following the detection of trophoblast cells in the lungs of deceased women. Bi-directional cell trafficking between mother and fetus is also altered in pre-eclampsia and has been suggested to contribute to the underlying etiology. FMM has been implicated in tolerance promotion, remission of auto-inflammatory disorders during pregnancy, or the development of autoimmune conditions post-partum. The underlying mechanism whereby the host immune system is modulated is unclear but appears to involve HLA class II molecules, in that incompatibility between mother and fetus promotes remission of rheumatoid arthritis, whereas feto-maternal HLA compatibility may assist in the post-partum initiation of scleroderma. Couples having a high degree of HLA class II compatibility have an increased risk for pre-eclampsia, while the occurrence of scleroderma and rheumatoid arthritis is greater in pre-eclamptic cases than in women with normal pregnancies, suggesting a long term autoimmune predisposition. Since pregnant women with pre-eclampsia exhibit significantly lower levels of maternally-derived micro-chimerism, the question arises whether pre-eclampsia and post-partum development of autoimmune conditions occur due to the failure of the grandmothers cells to adequately regulate an inappropriate micro-chimeric constellation.

## FMM and Pre-Eclampsia: Historical Observations

Granted that pre-eclampsia, a severe life-threatening disorder of pregnancy characterized by hypertension, proteinuria and organ failure is proposed to arise from dysfunctional placentation (1). It is hardly surprising that many key observations concerning feto-maternal cell trafficking and ensuing micro-chimerism were made in this context (2, 3).

In this regard, it is generally accepted that the first evidence of FMM was made at the turn of the nineteenth century by Georg Schmorl; a German pathologist in his examination of pregnant women who had succumbed to eclampsia (2, 4). Eclampsia is the fulminant form of pre-eclampsia where very severe symptoms are accompanied by seizures (1). In his report, Schmorl documented the occurrence of multi-nucleate trophoblast cells in the lungs of 14 out 17 cases with eclampsia. It is noteworthy that this feature was absent in 4 pregnant women who had died from other causes, suggestive that it may be linked to the underlying pathology of eclampsia (2, 4). Due to the potential role of the placenta as a key aetiological trigger in pre-eclampsia, the pivotal findings of Schmorl relating FMM to altered placentation proved to be worthy of more than passing interest and formed the basis of numerous other studies examining trophoblast deportation, the release of trophoblast micro-particles or even cell-free DNA from the placenta in cases with eclampsia or pre-eclampsia (5–7). Consequently, it was worthwhile reappraising Schmorl's widely-cited seminal report in translated form (4).

These were, however, not the only early reports suggesting that pre-eclampsia may be associated with altered feto-maternal cell trafficking. In 1905 Diehl postulated that pre-eclampsia may result from increased transfusion of incompatible fetal blood cells into the maternal compartment (2). This hypothesis fitted with the then current concept of pre-eclampsia being a form of *pregnancy toxemia*, triggered by toxins, possibly of placental origin during pregnancy (8). Further evidence supporting enhanced feto-maternal bleeding in pre-eclampsia was provided by epidemiological observations suggesting that the frequency of immunization due to Rhesus D incompatibility was greater in pregnancies affected by pre-eclampsia than in those with healthy deliveries (9). Since the Rhesus D antigen is expressed exclusively on cells of the erythrocyte lineage (10), such maternal immunization could not be due to the increased presence of placental trophoblast but would of necessity involve the increased presence of fetal red blood cells in the maternal circulation. This vital aspect was subsequently confirmed by the use of the then novel Kleihauer-Betke stain, which permitted the detection of fetal erythrocytes in maternal blood samples (11). This indicated that their proportion was indeed greater in pregnancies affected by pre-eclampsia (12). These data thereby provided further insight into the placental lesion in pre-eclampsia, indicating that it involved both increased trophoblast shedding, as well as leakage of fetal blood cells across the villous barrier (2, 3).

## FMM: The Question of Fetal Cells Persistence

Possibly one of the most exciting subsequent developments was the detection of fetal leucocytes in mitogen stimulated maternal blood samples, wherein male fetal cells could be detected by the presence of a Y chromosome (13). While this finding was confirmed in a number of ensuing studies, these did highlight the possible longevity of such fetal leucocytes as they could be detected for a considerable period post-partum (14–16). Indeed, the question arose whether such persisting fetal cells could contribute to the presence of male leucocytes in maternal blood samples from pregnancies with a female fetus (14–16).

In a landmark finding the research group of Diana Bianchi reported on the detection of circulatory fetal cells with stem cell-like characteristics for a period of almost three decades post-delivery (17). Subsequent studies have revealed that fetal cell persistence is a frequent event occurring both in mouse and man, and that this affects a variety of tissues including the brain (18–20). As we shall observe in the continuation of this discourse, the longevity of trafficking fetal cells provided the impetus for a number of other investigative routes, particularly with regard to the development of autoimmune conditions since women are more prone to develop them post-partum (21).

## FMM: The Quest for Non-Invasive Prenatal Diagnosis Leads to New Developments

The advent of amniocentesis and karyotyping revolutionized obstetrical practice by facilitating the prenatal assessment of fetal chromosomal anomalies, such as trisomy 21 occurring in Down syndrome (22). A caveat of such invasive prenatal diagnostic procedures, especially that of chorionic villous sampling performed earlier in gestation, was the risk of injury to mother and potential loss of the unborn fetus (23–25). Consequently, the need was voiced for safe efficacious alternatives, thereby fuelling the quest for methods permitting non-invasive prenatal diagnosis. It was therefore a foregone conclusion that the prior reports of FMM would provide the basis for the development of such novel prenatal tests (26). Thus, most of these early studies focussed either on the detection of fetal cells in maternal blood while a few examined for the presence of trophoblast cells in the cervix of pregnant women (27). Due to their scarcity, fetal cells in maternal blood had to be enriched with most centers using either flow cytometric or magnetic cell sorting approaches (28).

The fetal erythroblast, also termed nucleated red blood cell, emerged as the target cell of choice for fetal cells in maternal blood-based strategies. This was largely due to its abundance in the fetal circulation, its short half-life that precluded any issue pertaining to longevity, as well as the availability of suitable markers for enrichment (CD71) and identification (fetal and embryonic hemoglobin) (26, 28). By the use of multi-color FISH (fluorescent *in-situ* hybridization) early studies suggested that it may be possible to detect the most common fetal aneuploidies by this approach (28, 29), while the use of single cell PCR permitted the detection of Mendelian disorders i.e., hemoglobinopathies (30, 31). These encouraging results promoted the assessment of this challenging route in two independent NIH and EU funded studies (32, 33). Unfortunately, both of these multicenter studies revealed that the paucity of fetal erythroblasts in maternal circulation rendered their use impractical in daily clinical routine since the required level of specificity and sensitivity could not be attained (32, 33).

During these explorations our group did make two pertinent observations with regard to feto-maternal cell trafficking in preeclampsia. In the first study, by the use of male singleton pregnancies and multi-color FISH for the X and Y chromosomes we could irrevocably demonstrate a significantly increased presence of male fetal erythroblasts in the maternal circulation in cases that manifest pre-eclampsia when compared to matching healthy control pregnancies (34). Furthermore, in an examination of maternal blood samples collected from an at-risk cohort we observed an increase in male fetal erythroblasts in samples from cases that subsequently developed pre-eclampsia (35). Therefore, these results confirmed that the underlying placental lesion in pre-eclampsia facilitated leakage across the usually tight feto-maternal barrier and that this defect was an early event in the course of this disorder occurring prior to clinical manifestation of symptoms (2, 3).

## FMM: Not Restricted to Cells But Also Includes Cell-Free DNA

During the “NIH NIFTY” study that explored the use of fetal cells for non-invasive prenatal diagnosis the discovery of fetal cell-free DNA (cfDNA) in maternal plasma or serum was reported by Dennis Lo and colleagues in Oxford using Y chromosome specific PCR (36). The basis for the Oxford study were reports on the presence of tumor-derived cfDNA in cancer patient sera (37, 38). In an extension of their original findings they observed that maternally-derived cfDNA fragments could be detected in cord blood samples indicating that FMM was not restricted to the cross placental traffic of cells but could also involve cellular cfDNA fragments (39).

The use of real-time PCR permitted a rapid and precise assessment of the concentration of cfDNA in maternal blood samples. This indicated a progressive increase during pregnancy, peaking at term and ending with rapid clearance post-partum (40). Due to our interest in pre-eclampsia we performed several detailed investigations using qRT-PCR assays. These indicated that manifestation of pre-eclampsia was associated with a significant increase in the concentration of both fetal and maternally-derived cfDNA, which correlated with disease severity and being greatest in cases complicated with HELLP (hemolysis, elevated liver, low platelet) syndrome or eclampsia (41). Furthermore, we observed that when the blood samples were drawn early during pregnancy prior to the onset of clinical symptoms only the level of fetal cfDNA but not maternal cfDNA was elevated (42, 43). Since fetal cfDNA was determined to arise from the placenta this provided further evidence that the underlying etiology of pre-eclampsia involved placental lesions that occurred early in the cascade of events leading to clinical manifestation (3). It is still unclear whether or not elevations in the amount of fetal cfDNA contribute to the development of pre-eclampsia or other pregnancy-related disorders, such as preterm labor (44). The reason for this was that by its hyper-methylated status, fetal cfDNA could trigger an inflammatory response via TLR (toll like receptor) activation, thereby playing a crucial initiatory role (44). Unfortunately no clear data exists to support this enticing hypothesis (45). The question as to the source of elevated maternal cfDNA molecules was more complex to answer and took a rather unexpected turn when it was finally revealed to be derived from excessive generation of neutrophil extracellular traps (NETS) in pre-eclampsia (46, 47).

The advantage of cfDNA over fetal cells in maternal blood for prenatal diagnostic approaches quickly became apparent, particularly when examining for fetal genetic loci absent from the maternal genome, such as the Y chromosome for male fetuses, or the Rhesus D gene in Rhesus D pregnant women (48). Furthermore, the exploitation of the post-genomic advent of massive parallel sequencing permitted the reliable detection of fetal chromosomal abnormalities, such as trisomies, thereby ushering this long sought goal into clinical practice (48, 49).

## FMM: Rheumatoid Arthritis—Amelioration, Prevention or Promotion?

Amongst the autoimmune rheumatic disorders, rheumatoid arthritis probably responds most favorably to pregnancy in that many cases exhibit amelioration or remission whilst women affected by systemic lupus erythematosus frequently have poor pregnancy outcome characterized by high rates of pre-eclampsia or even fetal loss (50, 51).

Since the beneficial effect of pregnancy on rheumatoid arthritis disease status was determined not to be due to the action of immune-suppressive hormones, Lee Nelson and colleagues examined whether an HLA-based interaction between mother and fetus could contribute to disease improvement (52, 53). For their study they focussed on HLA Class II antigens due to the predisposing effect of certain alleles on rheumatoid arthritis incidence. In their analysis of 57 pregnancies from 41 women with rheumatoid arthritis remission was noted in 22 and amelioration in 12 instances. In 12 pregnancies no improvement of rheumatoid arthritis symptoms were noted (52). Their data indicated that a disparity in MHC class II alleles existed between mother and fetus in those pregnancies exhibiting a remission or improvement of rheumatoid arthritis symptoms. These were most pronounced for HLA-DRB1, DQA and DQB. Limited allelic differences were noted in the 12 rheumatoid arthritis cases where no improvement of symptoms was offered by pregnancy. This association between MHC class II disparity and disease activity was also evident in instances in women having successive pregnancies where rheumatoid arthritis symptoms were reduced in one instance but not the other. At the time that these studies were conducted, no conclusions could be drawn as to the operative mechanism although the question of trafficking fetal cells and FMM were raised (52).

Pregnancy may, however, not only afford some temporary respite from rheumatoid arthritis but may prevent or reduce the onset of this debilitating disorder post-partum (54). In order to examine whether pregnancy provided a protective effect against the development of rheumatoid arthritis, Guthrie and colleagues examined 310 women with newly diagnosed rheumatoid arthritis in comparison to 1,418 control women (54). This study indicated that the occurrence of rheumatoid arthritis was indeed lower in women who had been pregnant and delivered healthy babies by a factor of almost 40% when compared to non-parous women. It was furthermore observed that no reduction in the risk of developing rheumatoid arthritis was evident in women who had been pregnant but had not continued with the pregnancy until delivery (54). There was also no evidence for a cumulative protective effect offered by multiple deliveries. In contrast it appeared that the greatest protective effect offered by pregnancy was in a period of 5 years or less after delivery, and that this effect diminished significantly by 15 years post-partum. The authors proposed that the protective effect offered by a completed pregnancy may be attributable to persisting fetal cells. This could account for the diminished influence observed in pregnancies not completed to term, as cross-placental cell trafficking would be limited in these instances. The mechanism whereby micro-chimeric fetal cells would favorably modulate the maternal immune system to prevent rheumatoid arthritis onset may be by the provision of disparate MHC class I alleles as described above. Furthermore, in parous women deemed to be at high risk for rheumatoid arthritis due to the presence of two copies of HLA alleles, such as certain HLA-DRB1 loci, micro-chimeric fetal cells may be able to offset disease development by the provisional protective HLA molecules, such as the “DERAA” HLA-DRB1 locus. Evidence that such a mechanism may be operative is provided by the report of Pietsma and colleagues who observed that the protective “DERAA” locus in the form of a non-maternally inherited allele (NIMA) lead to a diminished risk for rheumatoid arthritis (55). The NIMA mechanism is discussed in more detail below.

On the other hand, FMM could assist with the initiation of an auto-inflammatory condition by provision of the necessary genetic background (56). An example of this is the “shared epitope” of certain HLA-DRB1 alleles associated with an increased incidence of rheumatoid arthritis (57). This “shared epitope” is a highly similar five amino acid peptide sequence contained in the HLA-DRB1^*^04 allele. In an elegant study Yan and colleagues set out to address whether FMM could contribute to the development of rheumatoid arthritis in women who were genetically “shared epitope” negative (58). In this study they examined 52 cases with rheumatoid arthritis and 34 healthy controls; 84% of cases and 92% of controls had at least one live birth. The mean age of cases was 51 and that of controls 42 years. By the use of “shared epitope” specific PCR assays they assessed the degree of microchimerism for such alleles in their study and control groups. Although some degree of “shared epitope” specific signals could be detected in both cohorts, the extent was significantly greater in cases with rheumatoid arthritis (58). In addition, the level of FMM for these “shared epitopes” was higher in rheumatoid arthritis cases than controls. Therefore, FMM, can contribute to the subsequent development of an autoimmune condition i.e., rheumatoid arthritis by the provision of necessary HLA haplotypes it via the inheritance of fetal cells or maternal cells during pregnancy (58).

## FMM: Cells From Grandma are Missing in Pre-Eclampsia

**Figure 1:**
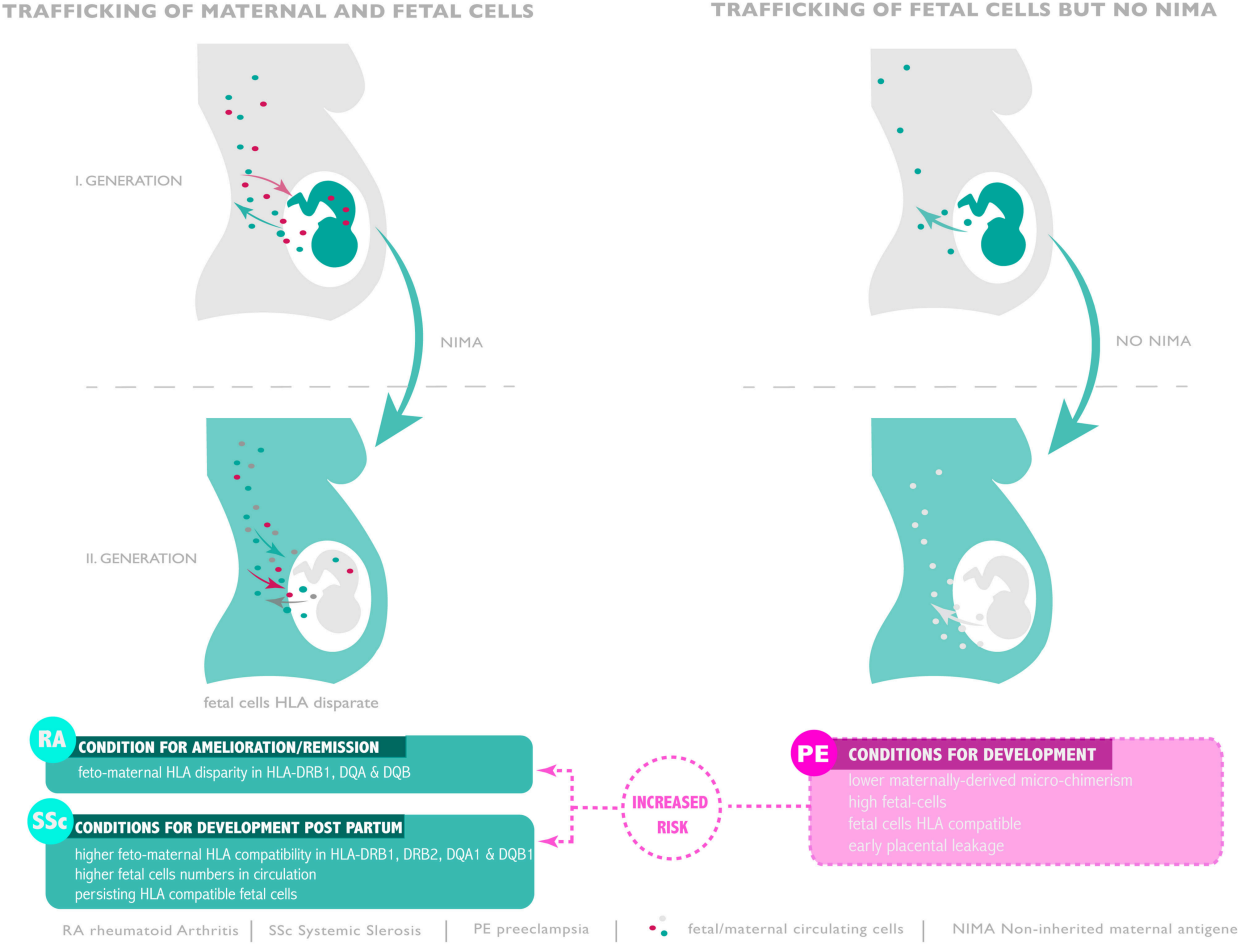
Microchimerism throughout generations and risk of pre-eclampsia, RA and SSc development. Compatible or disparate expression of HLA of non-inherited antigen present in subsequent generation seam to promote or ameliorate autoimmunity or favor pre-eclampsia.

During pregnancy, the pregnant woman exhibits a complex micro-chimeric phenotype, hosting cells from her current fetus, cells from previous pregnancies as well as cells from her mother (56, 59). Following the discovery of persisting fetal cells and their possible role in autoimmune conditions like systemic sclerosis, Maloney and colleagues investigated the behavior of trafficking maternal cells. Their examination, which employed HLA specific PCR assays as well as the use of XY FISH to detect female cells in male offspring revealed that cells of maternal origin can persist for numerous decades well into adulthood (59). In most instances maternal cells were HLA class I and II disparate from those of the host offspring (59). To study whether trafficking maternal cells play a role during pregnancy, Gammil et al. examined maternal blood samples collected in each trimester of pregnancy and post-partum (60). In some instances, they also had access to samples drawn prior to conception. By the use of quantitative PCR for specific HLA loci they were able to assess the extent of microchimerism attributable to trafficking maternal cells. In their examination of 86 maternal blood samples obtained from 27 healthy pregnant women with normal deliveries, no trafficking maternal cells were detected pre-conception or in samples obtained in the first trimester of pregnancy. On the other hand, such cells could be detected in 16% of second trimester, 29% of third trimester and 14% of post-partum samples. The degree of trafficking maternal cells microchimerism was greatest in samples collected close to term (60) (Figure 1). A startling finding made during this study was that no evidence of microchimerism due to trafficking maternal cells could be detected in any of the samples obtained from 20 pregnant women with manifest pre-eclampsia, in contrast to matching healthy control pregnant women, where such micro-chimeric cells could be detected in 30% of cases (60). This result could have profound implications since trafficking maternal cells are potentially important immune modulators due to their expression of NIMA (see below).

## FMM: The Role of NIMA

A number of early reports indicated that cross-placental cell traffic was not restricted to the fetus but could also be derived from the mother. Pioneering works performed by Owen and colleagues in 1954 observed that Rh-negative girls born from Rh-positive mothers develop very low amount of antibodies to Rh suggesting the existence of a tolerance mechanism to NIMA (61). Later and in the setting of solid organ transplantation tolerance, Claas et al. observed that 50% of the patients receiving several blood transfusions showed selective anergy to non-inherited HLA haplotypes (62). Similarly, Burlingham and colleagues describe that graft survival between siblings who are mismatched with the recipient for one HLA haplotype results in higher graft survival when the donor has maternal HLA antigens not inherited by the recipient than when the donor has paternal antigens not inherited by the recipient suggesting and highlighting an important role for NIMA (63). More works performed in this direction conclude that the risk of graft vs. host disease among NIMA-matched stem cell transplants is reduced, suggesting possible clinical benefits of NIMA-specific tolerance that persists in individuals through to adulthood (64, 65). Post-natal persistence of genetically foreign chimeric maternal cells in offspring was originally described in infants with severe combined immune deficiency. In a study of 121 infants with defective T and B lymphocyte development, 40% had engrafted maternal T cells and a similar proportion developed clinically apparent graft-vs.-host disease caused by anti-fetal-allo-immunity (66, 67). Mother to offspring transfer and persistence of maternal cells is likely an unavoidable by-product of a porous placental interface. In this scenario, post-natal persistence of NIMA-specific tolerance represents an expendable developmental remnant of immune suppressive mechanisms essential for *in utero* survival (68). Very interesting is the role of NIMA during fetal life: Kinder and colleagues showed that the developmental exposure to foreign maternal cells primes the expansion and accumulation of NIMA-specific immune suppressive regulatory T cells that help establishing better tolerance (68, 69). The persistence of those regulatory T cells could then reinforce fetal tolerance during the next generation pregnancies sired by males with overlapping MHC haplotype specificity, conferring a reproductive advantage.

Taken together, genetic fitness is not restricted to chromosome transmission but is expanded through vertical transfer and survival of tolerogenic cells that establish microchimerism in offspring favoring in return the preservation of NIMA. On the other hand, cross-generational reproductive advantages that preserve post-natal retention of microchimeric maternal cells may also perpetuate auto-inflammatory or autoimmune diseases in offspring (56).

## FMM: Systemic Sclerosis—Triggered by Persisting Fetal Cells?

Unlike rheumatoid arthritis where the risk is diminished post-partum, systemic sclerosis, an autoimmune disorder characterized by graft-vs.-host disease like symptoms has a strong predilection in women post-partum (21). It was therefore hypothesized that persisting fetal cells may play a role in initiation of systemic sclerosis (21). In order to address this possibility, a group of 40 women who had previously given birth to a son were recruited. Of these, 17 were affected by systemic sclerosis, 7 were sisters of affected cases and 16 were healthy matching controls. The degree of fetal cell persistence in the circulation was assessed by a quantitative PCR assay specific for the Y chromosome. This analysis indicated that the median level of male positive cells was significantly higher in the circulation of systemic sclerosis cases than in matching controls, with intermediate levels being recorded in the 7 siblings (21). Since rheumatoid arthritis remission or amelioration during pregnancy involves a disparity in HLA class II, the role of such an interaction between mother and fetus was examined also in the systemic sclerosis cases. This indicated that a high degree of compatibility between mother and child existed for HLA DRB1_1_, DRB1_2_, DQA1, and DQB1 in systemic sclerosis cases (21). Since this feature was not evident in the healthy controls group, it is possible that microchimerism due to persisting HLA compatible fetal cells may contribute to the development of systemic sclerosis post-partum (Figure 1).

## FMM: What is the Influence of Pre-Eclampsia on Incidence of Systemic Sclerosis or Rheumatoid Arthritis?

As our report on elevated trafficking of fetal cells in pre-eclampsia (34) was published at around the same time as that of persisting fetal cells in systemic sclerosis (21), we queried whether the incidence of systemic sclerosis was higher in women who had pre-eclamptic pregnancies (26). Unfortunately, we did not have the correct epidemiological data set at our disposal at the time and so this hypothesis lay resting for almost two decades to finally be addressed in two recent reports (70, 71). In the first van Wyk and colleagues examined whether a relationship existed between the incidence of pre-eclampsia and subsequent development of systemic sclerosis (70). In their study cohort (*n* = 103), the incidence of systemic sclerosis post-first delivery was in mean after 27 years. The authors also determined that systemic sclerosis occurred with greater frequency in women who had pregnancies affected by pre-eclampsia or fetal growth restriction. A limitation of this study was that no information was available concerning potential HLA compatibility, a facet which would have been most interesting to investigate; on one hand in view of previous findings reporting on FMM (21), on the other hand due to reports indicating that pre-eclampsia may occur with greater frequency in instances where a high degree of MHC compatibility for the HLA-DR, -DP, and -DQ alleles occurs between spouses (72). In the second study, which made use of the Danish national register from which the pregnancy outcome and co-incidence of systemic sclerosis for 778,758 women was obtained (71). This indicated that the occurrence of pre-eclampsia is associated with a 69% risk of developing systemic sclerosis. Once again, unfortunately no details concerning HLA types could be obtained.

In the instance of post-partum development of rheumatoid arthritis, the issue is somewhat more complex with a major difference existing with regard as whether the pregnancy was healthy or disturbed by pre-eclampsia. As discussed above, it is widely accepted that prior pregnancy has a beneficial effect on reducing the subsequent development of rheumatoid arthritis. Indeed, it has even been suggested that parity may serve as a vaccine to prevent post-partum rheumatoid arthritis, especially in the first 5 years after the last delivery (54). This is in stark contrast to what is observed if the pregnancy was affected by pre-eclampsia, where an alternate scenario emerges in that such a constellation may favor post-partum rheumatoid arthritis. Evidence of such a feature was initially obtained by epidemiological data mining of the Danish National Patient Register where a retrospective examination suggested that pre-eclampsia was associated with an increased risk for post-partum development of rheumatoid arthritis (73). This feature was confirmed in a subsequent more detailed examination of 55,752 pregnant women of which 169 developed rheumatoid arthritis during the follow up period (74). The incidence of pre-eclampsia was greater in the rheumatoid arthritis study group (6.5%) than in the control cohort (3.6%) suggesting that pre-eclampsia lead to an increased risk for rheumatoid athritis (R.H = 1.96). On the basis of these findings the authors argued that rheumatoid arthritis may be associated with a long pre-clinical phase prior to symptom manifestation, a feature which may affect pregnancy outcome and thereby promote pre-eclampsia, or that a shared predisposition for pre-eclampsia and rheumatoid arthritis may exist (74) (Figure 1). In an independent study Ma and colleagues examined whether adverse pregnancy outcome as measured by extreme (≤1,000 g) or very low birth weight (≤1,500), a common feature of severe pre-eclampsia, was associated with subsequent clinical manifestation of rheumatoid arthritis (75). In their study they examined 202 cases with rheumatoid arthritis and 1,102 controls. This analysis indicated that both extreme and very low birth weight had a 3- to 5-fold greater risk of rheumatoid arthritis, particularly for the rheumatoid factor positive form (75).

## FMM: The Pre-Eclampsia Conundrum and the Role of NIMA

The incidence of pre-eclampsia, particularly the severe form involving a defect in placenta development and spiral artery modification is associated with a high degree of HLA class II compatibility between spouses specifically for the HLA-DR, -DP, and -DQ alleles (72). Since trans-placental fetal cell traffic is enhanced in cases with pre-eclampsia (34), this would lead to a high level of microchimerism involving HLA class II compatible fetal cells of the type previously observed in cases with systemic sclerosis, autoimmune condition characterized by graft-vs.-host disease-like lesions (21). In this context it is worth noting that previous studies have suggested that pre-eclampsia exhibits traits of an autoimmune condition with placental features akin to graft-vs.-host disease (76). The question, hence, arises if a histo-compatible FMM state contributes to the etiology of pre-eclampsia. It is also plausible that the presence of this high grade pro-autoimmune microchimeric setting in pre-eclampsia, involving a greater than normal dosage of fetal cells, contributes to the subsequent increased risk for systemic sclerosis and rheumatoid arthritis occurring years later in an extended post-partum period (71, 74).

As pointed out above, microchimerism in a pregnant woman not only involves cells from the current fetus, but also that of any previous pregnancies as well as trafficking maternal cells the proband inherited from her mother (60). Due to their expression of NIMA, these trafficking maternal cells are important in inducing tolerance. A striking feature of pre-eclampsia is the apparent lack of trafficking maternal cells (60). It is therefore possible that the immunological effect of the microchimeric imbalance occurring in pre-eclampsia due to an influx of fetal cells may be further skewed by the apparent lack of trafficking maternal cells in the proband. Consequently, there is no mechanism in place to check or dampen the deleterious action of FMM derived haplo-identical or similar HLA alleles. Since pregnancy is an inflammatory process characterized by basal activation of circulatory neutrophils (77), this could in combination with a micro-chimeric aberrancy provide the necessary stimulus for the dysregulation observed in pre-eclampsia. In a similar manner, the combination of a lack or reduced level of trafficking maternal cells and high grade fetal microchimerism of disease associated HLA alleles could contribute to post-partum onset of rheumatoid arthritis or systemic sclerosis.

## Summary and Conclusions

While feto-maternal exchange in pregnancy was previously viewed as rare and innocuous event, a vast body of work in the interim has shown that it is a common feature of mammalian pregnancy being readily discernible from mouse to man. The effects of this exchange are far reaching and can persist into adulthood and possibly even old age. In this review we have highlighted the potential anomaly regarding FMM occurring in pre-eclampsia and how this could contribute to the etiology of this enigmatic disorder, but also to the development of autoimmune diseases years post-partum. Should an imbalance between trafficking maternal cells and newly acquired fetal microchimerism prove to be a pivotal trigger driving these developments, then it is highly likely that an altering of the microchimeric milieu could be used to modulate or steer the maternal immune system away from this destructive direction. Evidence that such a mechanism may be viable is provided by reports indicating that the incidence of pre-eclampsia is reduced in women who have received blood transfusions, an event known to lead to a microchimeric state (78). It could therefore transpire that we are on the cusp of new era in the field of microchimerism, whereby this phenomenon provides the basis for new cell-based therapies geared at immune modulation and if so, what better target than pre-eclampsia.

## Author Contributions

SH and SR wrote the manuscript. LV prepared the figure. SH, PH, SvB, LV, NT, IH, OL, and SR discussed and revised the manuscript.

### Conflict of Interest Statement

The authors declare that the research was conducted in the absence of any commercial or financial relationships that could be construed as a potential conflict of interest.
